# Morning and nocturnal serum melatonin rhythm levels in patients with major depressive disorder**:** an analytical cross-sectional study

**DOI:** 10.1590/S1516-31802012000300006

**Published:** 2012-07-12

**Authors:** Shahnaz Khaleghipour, Mohsen Masjedi, Hassan Ahade, Meersalahodin Enayate, Gholamreza Pasha, Farah Nadery, Gholamhossein Ahmadzade

**Affiliations:** I Doctoral Student, Department of Psychology, Science and Research Branch, Islamic Azad University, Khouzestan, Iran.; II PhD. Assistant Professor, Department of Immunology, Isfahan University of Medical Sciences, Isfahan, Iran.; III PhD. Full Professor, Department of Psychology, Alame Tabatabaei University, Tehran, Iran.; IV PhD. Assistant Professor, Department of Psychology, Science and Research Branch, Islamic Azad University, Khouzestan, Iran.; V MD, PhD. Associate Professor, Department of Psychiatry, Isfahan University of Medical Sciences, Isfahan, Iran.

**Keywords:** Melatonin, Periodicity, Depressive disorder, major, Serum, Depression, Melatonina, Periodicidad, Trastorno depresivo mayor, Suero, Depresión

## Abstract

**CONTEXT AND OBJECTIVE:**

The pineal gland is an adaptive organ that precisely regulates the biological rhythms of melatonin brain hemostasis. Variation in the regulation of melatonin rhythms is a likely cause of depressive disorder. The purpose of this study was to measure serum melatonin levels in patients with major depressive disorder (MDD) and normal control subjects.

**DESIGN AND SETTING:**

Analytical cross-sectional study at the industrial medical unit of the Iron Smelting Company of Isfahan, Iran.

**METHODS:**

The morning and nocturnal serum melatonin levels of patients and controls were measured using the enzyme-linked immunosorbent assay (ELISA) method. All data were assessed using variance analysis.

**RESULTS:**

The morning and nocturnal serum melatonin levels of depressed and healthy subjects differed (P ≤ 0.05). The nocturnal serum melatonin levels of depressed women were lower than those of depressed men (P ≤ 0.05).

**CONCLUSIONS:**

The findings of this study showed that the nocturnal serum melatonin levels in the depressed patients were lower than in the controls. Thus, the peak melatonin phase in the depressed patients was reached with delay.

**CLINICAL TRIAL REGISTRATION NUMBER:**

NCT01357083

## ABSTRACTO

**CONTEXTO Y OBJETIVO:**

La glándula pineal actúa precisamente regulando los ritmos biológicos de melatonina de hemostasia cerebral, como un órgano adaptativo. La modificación del ritmo de melatonina puede ser el motivo probable del trastorno depresivo. Este estudio se realizó con el objetivo de medir los niveles de melatonina entre los pacientes con trastorno depresivo mayor y los sanos.

**DISEÑO Y ESPACIO:**

Estudio analítico transversal- la unidad medicina laboral de empresa de Zob Ahan de Isfahán-Irán.

**MÉTODO:**

Los niveles de melatonina en suero día-noche se midó entre dos grupos (pacientes y sanos) utilizando el método de ELISA (Ensayo por inmunoabsorción ligado a enzimas). Todos los datos se hizo utilizando el análisis de la varianza.

**RESULTADOS:**

El nivel de melatonina en suero día-noche era distinto entre los deprimidos y los saludables (P ≤ 0.05). El nivel de melatonina en suero en las mujeres deprimidas fue menos que los varones deprimidos (P ≤ 0.05).

**CONCLUSIONES:**

Esta investigación muestra que el nivel de melatonina nocturna en los deprimidos ha sido menos que los controlados, pues el pico de fase de melatonina en los pacientes deprimidos alcanza con retraso.

## INTRODUCTION

Major depressive disorder (MDD) is the most severe type of depression, and it is termed a mood disorder. MDD is accompanied by different physical, emotional, cognitive and motivational signs. Extensive studies are currently investigating the etiology of this disorder. However, experts still use clinical signs to diagnose this disorder.^1^

Biological studies over recent decades have been investigating abnormalities in biological rhythms, neurotransmitter levels, receptor variation and endocrine levels in particular regions of the brain. Through these, the development and maintenance of MDD are becoming better understood than before. One of the main points in the biological trends relates to circadian rhythm and disturbances in this cycle, which cause mood disorders and irregularity in this bioclock, thereby leading to depression.^2^

The pineal gland provides precise regulation of the circadian rhythm of melatonin, which regulates brain homeostasis. Abnormal functioning of this gland gives rise to psychiatric disorders.^3^ The pineal gland produces melatonin from L-tryptophan amino acid, which is secreted into the circulation and cerebrospinal fluid (CSF) circulation.^4^ The synthesis rhythm and secretion of melatonin are produced 24 hours/day, endogenously available in the suprachiasmatic nuclei of the brain^5^ and, in some manner, they play role as an internal zeitgeber.^6^^,^^7^ The melatonin of the gastrointestinal tract is produced from serotonin. In this regard, serotonin is activated by the enzymes serotonin N-acetyl transferase and hydroxyindole-1-methyl transferase. Serotonin is converted into melatonin by the second of these enzymes.

Lerner^8^ was the first to study the physiological or behavioral effects of exogenous melatonin, on himself. He received 200 mg intravenously per day for five successive days, and subsequently he felt relaxed.^9^ His actions led to better knowledge of some of the positive and negative aspects of melatonin. In 1975, it was found that nocturnal plasma melatonin levels are 10 times greater than daytime levels,^10^ and that serum melatonin levels reach a peak between 11 pm and 3 am.^11^

Melatonin is responsible for regulation of sleep and awareness. It is related to sleep phase delay syndrome,^12^^,^^13^ protection against androgen oxidizing and prevention of aging.^14^^,^^15^ Excessive physiological levels of melatonin cause biological effects like daytime drowsiness, disorders of psychological and physical functioning^16^ and body temperature reduction.^17^ Melatonin secretion increases in the dark and it declines in daylight. Therefore, people show different reactions to light, which is under the influence of environmental and genetic factors. This dissonance can affect melatonin secretion.^4^ An increase in melatonin secretion is accompanied by a fall in consciousness and function, increased theta wave activity in the brain and increased drowsiness and excessive fatigue overnight.^18^ Reduction in melatonin secretion is accompanied by an increase in body temperature (hyperthermia), reduced propensity to sleep, reduced theta wave activity and reduced propensity to daily rapid eye movement sleep (REM sleep).^19^

During youth and early middle age, depression is more common because of certain tough conditions.^20^^,^^21^ These conditions cause biochemical changes and disturbances to biorhythms, including melatonin secretion. In elderly people, because of irregularity in some circadian rhythms and decreased activity in the suprachiasmatic nuclei (SCN) of the hypothalamus, melatonin secretion is seen to have inhibitory action.^2^^,^^22^ The psychiatric mechanisms associated with depression are induced by hyperarousal of the hypothalamic-pituitary-adrenal axis and are linked to the pineal gland. This process can change the peak of the melatonin phase. In addition, a decrease in serum melatonin levels can change the way in which the immune system functions in depressed patients. This facilitates the process of cancerous cell formation and tumor growth.^23^^,^^24^ Sekula et al.^25^ showed that an increase in serum melatonin rhythm level could produce MDD. However, Dolberg et al.^26^ demonstrated that “low melatonin syndrome” influenced the quality of sleep and cannot predict depression. A study on acute multiple sclerosis patients with concomitant MDD showed that the time of peak melatonin occurred 77 minutes later than in individuals without MDD.^27^

## OBJECTIVES

Given the conflicting results and the fact that the positive and negative roles of melatonin in creating depression are unknown, the aim of this study was to compare morning and nocturnal serum melatonin rhythm levels in patients with MDD. The second aim was to measure morning and nocturnal serum melatonin levels in depressed and healthy men and women.

## METHODS

### Patients

This was an analytical cross-sectional study. A group of depressed patients and a group of healthy control subjects were selected by convenience sampling. The individuals were chosen from among patients attended at the industrial medical unit of the Iron Smelting Company of Isfahan, Iran. These individuals underwent a medical examination and then gave responses in the Beck depression inventory (BDI-II). Those who obtained a depression score greater than 20 were included in the depressed group. Among this group, 50 patients were chosen. Likewise, among the individuals who obtained a depression score less than 9, 50 were chosen for inclusion in the healthy control group. The control group was chosen to match the depressed patients in terms of educational, social, occupational and economic situations. All of the subjects were interviewed psychiatrically, and the presence of depression disorder was confirmed on the basis of the Diagnostic and Statistical Manual of Mental Disorders (DSM) criteria. It should be noted that eight out of the 50 depressed patients were excluded from the study because they were suffering from anxiety disorders based on the Structured Clinical Interview for the DSM (SCID). The exclusion criteria consisted of the presence of drug consumption, use of narcotic substances, physical and psychosomatic disorders, stressors and malfunctioning of the thyroid gland.

### Beck depression inventory (BDI)

The BDI is a 21-item self-reporting scale that is designed to measure the signs of depression in the general population. It assesses the primary symptoms used to make a clinical diagnosis of depression. The revised draft of the scale, namely, BDI-II, presents greater concordance with the DSM than shown by the first draft and is based on cognitive theory. It covers all of the elements of depression.^28^ In relation to the revised draft, Steer et al.^28^ have reported that the coefficient of internal stability ranges from 72% to 92%, with a mean of 86%, and that the alpha coefficient for patients is 86% and for normal healthy controls is 81%.^28^

### Quantitative determination of melatonin levels

The examinees were asked not to take any medicine for one week before the melatonin assay. Venous blood (5 ml) was taken from both groups at 8:00 am on a particular day. At the end of the same day, at midnight, blood samples were again taken from the subjects under dim light in the laboratory of the Motaharea hospital of the Iron Smelting Company. The blood samples were centrifuged, and their sera were separated within 30 minutes. The sera were incubated at -70 °C. Serum melatonin levels were assayed using an enzyme-linked immunosorbert assay (ELISA) kit (cat no. RE54021; IBL, Hamburg, Germany) with the following characteristics: sensitivity, 1.6 pg/ml; intra-assay coefficient of variation (CV), 3.0-11.4%; inter-assay CV, 4-19.3%).

To extract the solid phase, the extraction columns were first placed in the glass tubes for the column conditioning. Then, 2 x 1 ml of undiluted methanol was added to the column; the solvent was passed through the column by means of centrifugation, and the eluate was discarded. Then, 2 × 1 ml of double distilled water was added to the columns, and the solvent was allowed to pass through the column by means of centrifugation, and the eluate was discarded. The extraction columns were then placed in the glass tubes for the sample application, and 0.5 ml of standards, controls and samples were added to the columns. Then, 2 × 1 ml of 10% methanol in double distilled water (v/v) was added to the columns, and centrifuged and the eluate was discarded. The extraction columns were then placed in new glass tubes and 1 ml of undiluted methanol was added to the columns, and the columns were removed from the tubes. The solution collected from the tubes was then used for the next steps.

The methanol was then evaporated to dryness, using the evaporator centrifuge. The samples were reconstituted with 150 µl of double distilled water and 50 µl of each extracted standard, extracted control and extracted sample were added to the respective wells of the ELISA plate. Then, 50 µl of melatonin biotin was added to each well, and 50 µl of melatonin anti-serum was added to each well. Finally, the plate was covered with adhesive foil. The plate was shaken carefully, and incubated for 14-20 hours at 2-8 °C. Following incubation, the plate was washed three times with 250 µl of diluted assay buffer, and 150 µl of freshly prepared enzyme conjugate was added to each well, and the plate was incubated for two hours at room temperature. After discarding the incubation solution, the plate was washed three times with 250 µl of diluted assay buffer. Then, 200 µl of para-nitrophenyl phosphate (PNPP) substrate solution were added to each well, and the plate was incubated for 20-40 minutes at room temperature. Finally, 50 µl of PNPP stop solution were added to each well, and the optical density (OD) was measured with a photometer at 405 nm (reference wavelength: 600-650 nm).

### Statistical analyses

All statistical analyses were performed using the Statistical Package for the Social Sciences (SPSS) 16.0 for Windows. Chi-square tests were performed on the categorical variables. T tests were carried out on the variables of age and depression. Univariate analysis was also carried out on the morning and nocturnal serum melatonin levels. A P-value of less than 0.05 was considered significant.

## RESULTS

The depressed patient group comprised 28 women and 14 men, and the healthy subject group comprised 26 women and 24 men.

Table 1 shows the mean ages and depression scores for the depressed patients and their matched controls. The patients were between 22 and 47 years old, with a mean age of 37.83 ± 7.70. The healthy subjects were between 24 and 48 years old, with a mean age of 36.64 ± 6.82. The mean depression score of the healthy subjects was 2.46 ± 2.38. There was a significant difference in depression scores between the depressed patients and the healthy controls (P £ 0.05).

**Table 1.** Demographic features of the depressed and healthy subjects

	Depressed group	Control group	P
Number	42	50	
Gender			
Male/female	(14/28)	(24/26)	0.113
Age (range) mean	(22-47) 37.83 ± 7.70	(24-48) 36.64 ± 6.82	0.787
Depressive score	27.57 ± 7.15	2.46 ± 2.38	0.05

The results from the univariate analysis of variance showed that the morning serum melatonin levels of the depressed and healthy subjects differed (F = 23.93; P < 0.001; df = 1). Table 2 shows that the mean morning serum melatonin levels were higher in the depressed patients than in the controls. The results also revealed that there was no significant difference between the genders regarding the morning serum melatonin levels, such that the morning serum melatonin levels of women and men did not differ (Table 2).

**Table 2.** Serum melatonin levels in the depressed patients and controls

		Depressed group	Control group	P
Morning melatonin levels	Female	14.28 ± 3.28	10.62 ± 2.52	0.228
	Male	18.81 ± 96.2	11.27 ± 4.34	
	Total	16.45 ± 3.13	10.13 ± 3.24	0.001
Nocturnal melatonin levels	Female	37.48 ± 7.11	62.29 ± 4.10	0.001
	Male	48.85 ± 11.66	73.39 ± 9.96	
	Total	41.27 ± 10.29	67.42 ± 16.17	0.001

The findings from the analysis of variance on the nocturnal serum melatonin levels demonstrated that there were significant differences between the groups regarding the nocturnal serum melatonin levels (F = 60.92; P £ 0.001; df = 1), such that the nocturnal serum melatonin levels in the depressed patients were lower than in the controls (Table 2). The data also showed that there was a significant difference between the genders regarding the nocturnal serum melatonin levels, such that the nocturnal serum melatonin levels of the women differed from those of the men (F = 22.51; P £ 0.001; df = 1). This meant that the nocturnal serum melatonin level of the women were lower than those of the men (Table 2).

**Figure 1.**
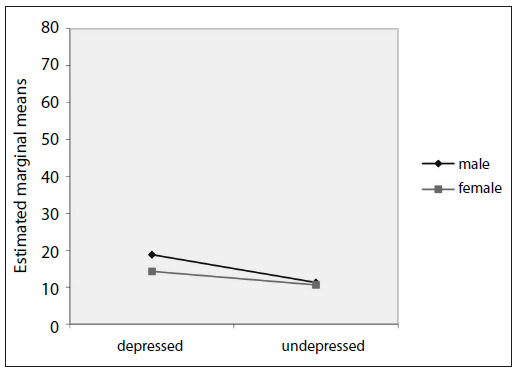
Estimated marginal means of morning serum melatonin levels. P-value between groups = 0.129.

**Figure 2.**
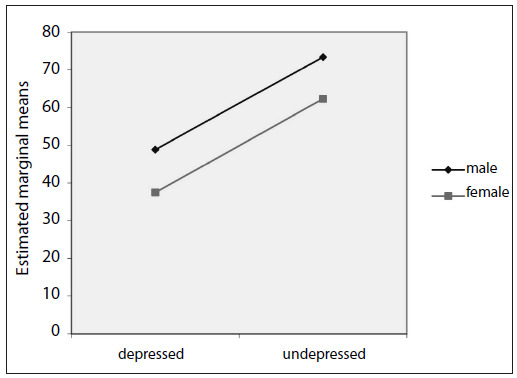
Estimated marginal means of nocturnal serum melatonin levels. P-value between groups = 0.368.

The results from analysis of variance on the morning and nocturnal serum melatonin levels revealed that there were no significant differences between the integrated effects of group versus gender for the morning and nocturnal serum melatonin levels (Figures 1 and 2).

## DISCUSSION

The current study aimed to compare the morning and nocturnal serum melatonin rhythm levels in subjects with MDD. The findings from the study showed that the nocturnal serum melatonin levels were significantly lower in the depressed patients, than in the controls **(**Table 2**)**. These findings disagree with the data of Sekula et al., who found that the nocturnal serum melatonin levels in depressed patients were higher and that these patients showed phase-shifted melatonin levels. The differences between these studies may be due to the use of antidepressants,^29^ severity of depression, use of beta blockers or hormonal drugs, advanced age or factors relating to light and season.

It should be noted that serum melatonin secretion levels vary according to the season of sampling. For instance, they change in the spring and winter due to the atmospheric conditions.^30^ Another important finding from the present study was that the morning serum melatonin levels were significantly higher in the patients with MDD than in the controls (Table 2). These results are inconsistent with the results of Akpinar et al., who found that there was no difference in morning serum melatonin levels between the depressed and control subjects. We do not yet know the reason for the high phase of morning melatonin. Nevertheless, it would appear that high levels of morning serum melatonin, associated with disorders in the temporal region of the brain, cause changes to rapid eye movement sleep within the nightly sleep rhythm of depressed patients.^31^ Accordingly, the differences between studies may reflect individual differences, insufficient nightly sleep or nocturnal awakenings. Most subjects who have nocturnal awakenings suffer from depression and have poor functioning in the mornings.^32^ They have also higher levels of morning serum melatonin than the nocturnal levels, and the circadian rhythms of cortisol, temperature and melatonin reach their maximum levels with a delay of one to three hours.^33^ We also investigated the serum melatonin levels comparatively between the men and the women and found significant differences in serum melatonin levels between them. The nocturnal serum melatonin levels were also significantly lower in the women than in the men (Table 2). The reason why depressed women^34^ have lower levels of serum melatonin than men do is that women are more vulnerable to bio-psycho-social factors.^35^

## CONCLUSIONS

Our data showed that the nocturnal serum melatonin levels in the depressed patients were lower than in the controls. Furthermore, the peak melatonin phase in the depressed patients was reached with a delay, compared with the controls. These findings suggest that melatonin deficiency may be among the factors implicated in occurrences of depression in patients with MDD. Nevertheless, the conflicting results demand further studies in order to seek better therapies for depressed patients with regard to serum melatonin rhythm levels. Likewise, further studies are needed in order to measure the corticotrophin-releasing hormone levels and personal characteristics such as sleep chronotype. Finally, factors that are effective in relation to melatonin secretion levels and psychiatric disorders can increase our understanding of the biological basis of these disorders, so that better solutions for treating depression can be found.
